# Errors in AI-Transformed Patient-Centered Mental Health Documentation Written by Psychiatrists: Qualitative Pre-Post Study

**DOI:** 10.2196/78351

**Published:** 2026-04-29

**Authors:** Pelin Ozkara Menekseoglu, Mareike Weibezahl, Mats Ellingsen, Jarl Sterkenburg, Anna Kharko, Stefan Hochwarter, Julian Schwarz

**Affiliations:** 1 Department of Psychiatry and Psychotherapy, Center for Mental Health, Immanuel Hospital Ruedersdorf, Brandenburg Medical School, Seebad 82/83, Ruedersdorf, 15562, Germany, 49 3363883501, 49 3363883502; 2 Faculty of Health Sciences Brandenburg, Brandenburg Medical School, Neuruppin, Germany; 3 Department of Computer Science, Norwegian University of Science and Technology, Trondheim, Norway; 4 Department of Women’s and Children’s Health, Uppsala University, Uppsala, Sweden; 5 HEALTH - Institute for Biomedical Research and Technologies, Joanneum Research Forschungsgesellschaft mbH, Graz, Austria

**Keywords:** open notes, large language models, psychiatric documentation, patient-centered communication, artificial intelligence, clinical safety

## Abstract

**Background:**

Patients’ digital access to their personal health data is becoming increasingly common worldwide. However, medical documentation often contains technical language and sensitive information, which can lead to potential misunderstandings and distress among patients. These issues may be particularly impactful in mental health contexts. Large language models (LLMs) offer a promising approach by transforming clinician-generated health notes into language that is more patient-centered, nonmedicalized, and empathetic. However, risks related to accuracy and clinical safety have not been adequately investigated in psychiatry.

**Objective:**

This study aimed to qualitatively analyze the errors introduced by LLMs when transforming notes written by psychiatrists into patient-facing formats. It also highlights the implications for clinical communication and patient safety.

**Methods:**

Clinical notes (n=63) written by 19 psychiatrists in an outpatient treatment setting were collected, anonymized, and translated from German to English by humans. OpenAI GPT-3.5 Turbo was used to develop a preprompt that transformed these notes into a patient-centered, lay-readable form through an iterative process. Three psychiatrists qualitatively analyzed the LLM-revised documentation using Kuckartz content analysis. They compared the preconversion and postconversion notes to systematically identify and categorize LLM-induced errors.

**Results:**

Five categories of clinically relevant errors were identified: (1) clinical misinterpretations, particularly in critical assessments such as suicidality, where nuanced terminology was oversimplified or inaccurately represented; (2) attribution errors, where behaviors or roles within family dynamics or interactions were incorrectly attributed to different individuals; (3) content distortion errors, which were characterized by speculative additions, emotional exaggerations, and inappropriate contextual assumptions; (4) abbreviation and terminology errors, which resulted from inaccurate expansions of medical abbreviations and terms; and (5) structural and syntax errors, which resulted in ambiguity, particularly when the original notes were brief or bulleted. Despite significant improvements in the readability and overall linguistic fluency of the converted notes, these errors occurred.

**Conclusions:**

LLMs have the potential to transform psychiatric notes into patient-friendly formats. However, critical errors remain prevalent and can impair clinical judgment, understanding of patient circumstances, clarity of medication regimens, and interpretation of clinical observations. To safely integrate artificial intelligence–generated documentation into psychiatric care, clinician oversight and targeted model refinement are essential. Future research should explore strategies to mitigate these errors, assess their comprehensive clinical impact, and incorporate patient and provider perspectives to ensure robust implementation.

## Introduction

Patients’ digital access to information about their own health, such as medical findings, has increasingly become common internationally, with an aim to promote transparency and patient-centered care. In several health care systems, such as those in the United States, United Kingdom, and Scandinavian countries, patients even have access to clinicians’ treatment notes, known as “open notes” [1,2]. Studies show that access to patients’ own open notes can improve communication, participation in the health care, and adherence to the treatment process. This benefits both patients and caregivers [3-5]. However, as clinical notes are generally written to meet the information needs of clinicians, using highly technical medical language and abbreviations, difficulties persist. Although written to document key aspects of the patient’s health and treatment, these notes can be difficult for patients to understand and may increase the risk of misinterpretation or confusion [6]. In psychiatry, particular concerns arise from sharing medical records due to the sensitive nature of psychiatric information [7]. Such data can provoke feelings of anxiety, distress, confusion, or stigmatization among patients [8-11].

To address this challenge, there is growing interest in using artificial intelligence (AI) to create more patient-centered language for medical documentation. AI has the potential to convert complex medical documentation into clear language and formats that can be easily understood by patients, enabling better quality care [12]. Large language models (LLMs), a class of generative AI systems, are capable of processing and generating large amounts of unstructured natural language text. Such models hold promise for streamlining the creation of patient notes, helping patients navigate the health care system, and enhancing clinical decision-making processes [13]. Studies examining the use of LLMs to transform medical notes into more patient-friendly language have been conducted in several countries, including the United States [14], Switzerland [15], and Germany [16]. It has been shown that LLMs can improve readability and comprehensibility, making medical summaries more accessible to patients [17]. Research findings on patients using LLMs to interpret open notes indicate that all models used have the capacity to answer patient questions to a certain degree. Nevertheless, the use of these models is found to be variable in accordance with model type, the configuration of the prompt, and the perspective of the evaluator [18]. Concerns have also been raised regarding LLMs, with one such concern relating to content awareness. This is also critical in treatment planning and can lead to uncertainty [19]. Furthermore, the implementation of electronic health records has been associated with unintended outcomes such as redundant entries, incorrect use of templates, misplacement of documents in patient charts, and inconsistent content. These electronic health record–related errors can compromise data accuracy, potentially jeopardizing patient safety and reducing quality of care [20]. As the adoption of LLMs in medical documentation continues to increase, and their role expands to include text modification, concerns have emerged about their applicability due to errors, such as fabricated information and omission of relevant content, highlighting the need for careful inspection and oversight [21]. However, research on the use of LLMs in psychiatry is limited, particularly regarding the types of errors caused by LLMs and strategies for addressing them.

As patient access to mental health data, including open notes, has expanded in recent years, developing solutions to enhance clinical documentation and prevent misunderstandings or conflicts arising from patients reading their notes has become highly relevant [22]. Thus, especially in psychiatry, it would be a significant advancement and time-saver for clinicians to have LLM-based tools supporting the creation of clinical notes [23]. At the same time, careful evaluation is essential to determine whether and to what extent LLMs introduce errors into human-authored documentation, ensuring that AI-based tools enhance, rather than compromise, the quality and clarity of personal health data in mental health.

This study aims to address this gap by exploring and systematically categorizing the types of errors that occur when LLMs are used to revise clinician-authored mental health notes, which are intended to make them more accessible and readable for patients.

## Methods

### Study Design

**Figure 1.**
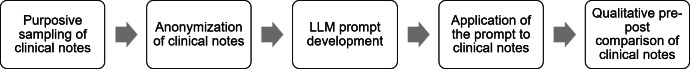
Methodological steps for collecting, preparing, and analyzing clinical notes. LLM: large language model.

This study is part of a broader investigation evaluating patients’ digital access to their mental health data, including the use of LLMs to enhance the patient-centeredness of open notes. While another part of the project focused on refining prompts iteratively to optimize the patient-centeredness of LLM-modified notes, this study examines the types of errors that may emerge from a psychiatric perspective as a result of LLM intervention. Due to the exploratory and descriptive nature of the study, a qualitative approach was selected. To capture only errors introduced by the LLM rather than errors already present in the original documentation, psychiatrists precisely compared each original (preconversion) note with its corresponding LLM-modified (postconversion) version. The analysis used Kuckartz qualitative content analysis methodology [24], using an inductive approach to identify and categorize clinically relevant and frequently occurring errors resulting from the conversion process. Figure 1 shows the key methodological steps in this study.

### Ethical Considerations

Ethics approval from the Ethics Committee of the Brandenburg Medical School (MHB) was obtained (reference number: E-01‐20210727). The study was registered with the German Clinical Trial Register (DRKS00030188).

The study used fully anonymized routine health record data. In accordance with applicable regulations, the requirement for informed consent was waived by the ethics committee, as no identifiable personal data were processed and no reidentification of individuals was possible.

All data were handled in compliance with relevant data protection regulations. Data were anonymized prior to analysis, and strict measures were implemented to ensure confidentiality and data security throughout the study.

No financial compensation was provided, as the study did not involve direct participant contact.

### Study Setting

The study used clinical documentation from 3 outpatient treatment units for patients with severe mental health conditions (referred to as “psychiatric institute ambulances”) at the Department of Psychiatry and Psychotherapy at Immanuel Hospital Rüdersdorf in Brandenburg, Germany. Patients who have access to this service must have a specific diagnosis, such as bipolar disorder, severe depressive episodes, or psychotic spectrum disorders (see [25] for a complete list of included International Classification of Diseases, 10th Revision diagnoses). The outpatient unit offers team-based, multiprofessional treatment with as many contacts as necessary. On average, patients have 3.2 treatment contacts with a team member in the psychiatric institute ambulance each quarter of the year [26]. At least one of these contacts must be with a psychiatrist. Each treatment contact is documented in the form of a free-text note within the hospital information system. This note contains crucial information about the treatment contact for the person documenting it, the treatment team, and for reimbursement reasons.

### Collection and Preparation of Clinical Notes

Clinical notes originally authored in January 2024 by psychiatrists were selected during the same month from the hospital information system of the study centers. Each note could contain the details of an outpatient appointment, including symptoms, findings, diagnoses, medical history, treatment decisions, and planned interventions. It was imperative that this definition be applicable to all the notes in the sample. These were not open notes intended for patient access but rather conventional clinical documentation written solely for professional purposes. Following the principle of contrastive sampling in qualitative research [27], we purposefully selected notes with diverse characteristics. The sample included both brief and detailed notes, varying in structural complexity, and written by a broad range of practitioners (n=19) with different levels of professional experience (including residents and senior physicians) to capture a heterogeneous set of documentation styles and influences. Exclusion criteria included notes written during inpatient treatment or in emergency situations; notes consisting solely of structured fields such as medication lists, diagnosis codes, or checklist-style symptom assessments without narrative text, duplications; notes that are entirely administrative in nature (eg, appointment scheduling or prescription renewals); and notes that cannot be fully anonymized or lack the clinical context necessary for analysis.

A total of 107 clinical notes were subjected to screening. Notes that met the predefined inclusion criteria were iteratively included. In the subsequent step, all notes were manually anonymized by replacing personal identifiers, such as names, locations, and references to individuals, with fictitious information to prevent any possibility of identifying specific patients, while patient gender was preserved when explicitly stated to maintain the context. The anonymized notes were then translated into English using an offline version of the LibreTranslate software [28]. To ensure accuracy and preservation of meaning, the translations were independently reviewed in accordance with the 4-eyes principle by 2 professionals (PÖ and JS) with experience in psychiatry, who independently verified each translation. To ensure semantic and clinical equivalence, each reviewer independently compared the English translation with the original German note.

Special attention was given to the correct translation of medical abbreviations. Abbreviations used in the original German notes were carefully adapted to appropriate and comparable English equivalents, such as “Hausarzt” to “GP”(general practitioner) or “TR” to “therapeutic range.” In cases of uncommon or ambiguous abbreviations, decisions were made in consultation with a tandem of native German and English speakers to ensure contextually accurate rendering. During this step, no changes were made to the interpretation or content. The correct handling of abbreviations was a central focus throughout the process, as transforming medical shorthand into nonmedicalized, comprehensible language is a key objective of the LLM-based note revision.

### LLM-Based Conversion of Clinical Notes

We tested and refined multiple prompts using various LLMs (eg, Mistral, Llama, ChatGPT, and Copilot) to convert clinician-authored notes into patient-centered versions. During the pretest phase, study team members with clinical expertise evaluated these models’ outputs. Based on their subjective assessments, GPT-3.5-Turbo-0125 was selected because it produced results that better aligned with the clinical meaning from the psychiatrists’ perspective. Decoding parameters were set as follows: temperature=0, top-p=1, and max tokens=512. Each note was run once, with temperature=0 chosen to minimize variance across outputs. The final prompt included instructions to revise clinical notes into empathetic, comprehensible, and nonjudgmental formats while strictly preserving the original content and maintaining the third-person singular perspective. To prevent bias, the finalized prompt was applied unchanged to the 63 notes analyzed in this study. This prompt was developed and optimized on a separate set of clinical notes that were not included in the final dataset.

After iterative evaluations by 3 psychiatric researchers (PÖ, MW, and JS) between March and December 2024, the following prompt was finalized and applied to optimize the clinical notes in this study:

*Your task is to rewrite the following doctor’s note in empathetic language. In other words, adapt the note to be patient-friendly, focusing on clarity and expressing the content sensitively. Since the note is intended for the patient, it is important to write it in an easy-to-read manner. The purpose of the translation is to enable patients to read and understand psychiatric notes without much effort or knowledge of medical terms. This also helps reduce the gap between patients and doctors and increases overall trust in health professionals. The third-person singular perspective must be maintained. If the patient’s gender is unclear, use the singular “they.” The note must contain only patient information that is explicitly stated. The note should only include judgments that are explicitly stated. Use the original wording of assessments where possible.*

### Qualitative Data Analysis

#### Overview

The research team received each original clinical note along with its LLM-generated counterpart for qualitative comparison. Differences between the 2 versions were analyzed, and various types of errors in the AI-generated notes were identified, described, and systematically coded using Kuckartz method of qualitative content analysis, supported by MAXQDA software (VERBI Software) [29]. The analysis followed an inductive approach, allowing error categories to emerge directly from the data rather than applying predefined classifications.

The coding process was conducted in multiple iterative cycles. During the first cycle, open coding was used to identify initial patterns of LLM-related errors, including language distortions, factual inaccuracies, and structural modifications. In the second cycle, these initial codes were refined and grouped into broader thematic categories, such as clinical misinterpretations and attribution errors. These categories were developed through consensus among the research team. The refinement process involved repeated discussions and re-evaluations to ensure conceptual clarity and consistency.

#### Validation of the Developed Error Typology

**Figure 2.**
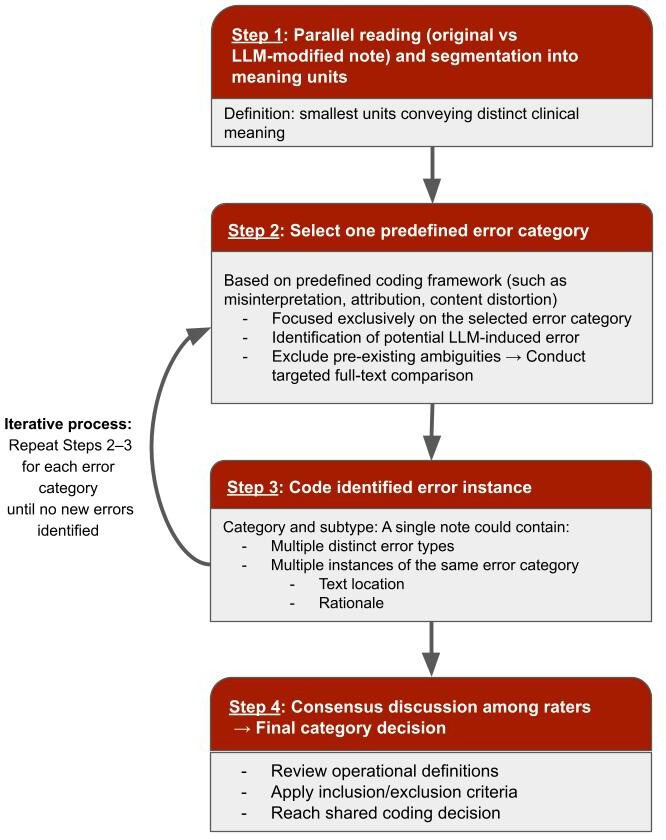
Iterative workflow for identifying LLM-modified errors. LLM: large language model.

To assess the validity of the identified error typology, we systematically described the analytical process of error detection (Figure 2) and developed a coding guide (see Multimedia Appendix 1).

### Quantitative Data Analysis

Finally, a quantitative error analysis was performed on the 20 notes. The purpose of reporting these findings is solely for descriptive; they should be interpreted as characteristics of the sample notes rather than as estimates of the overall effects of LLM use in psychiatric documents. To enhance the rigor of the analysis, interrater reliability was assessed by having multiple researchers (JS, MW, and PÖ) independently code a subset of the data and calculate Krippendorff α for this sample. Discrepancies were resolved through discussion, and consensus was used to finalize the coding framework.

## Results

### Sample

A total of 63 clinical notes and 63 corresponding LLM-transformed notes were analyzed and represent the sample of this study. The original notes were written by 19 psychiatrists: residents (n=5; 26.3%), specialists (n=9; 47.4%), and consultants (n=5; 26.3%). A total of 52% (n=10) of the psychiatrists were female participants. The original notes refer to 63 patients, aged 21 to 91 years (mean 50, SD 18.2), 73.1% of whom were female participants.

Original notes ranged from 60 to 1393 characters and from 9 to 223 words, corresponding to 13 to 300 tokens. After LLM conversion, the documentation length increased by 42.23% in characters, 39.29% in words, and 29.61% in tokens.

Table 1 presents the basic characteristics of the original notes and their corresponding LLM-transformed versions.

**Table 1.** Characteristics of original (N=63) and large language models–transformed (N=63) clinical notes.

Parameter	Original notes, mean (SD)	LLM^a^-transformed notes, mean (SD)
Number of characters (including spaces)	561.4 (289.1)	798.4 (369)
Tokens	125 (63.9)	162 (74.3)
Number of words	87.5 (47.7)	121.8 (59.3)

a LLM: large language model.

### Interrater Reliability

To assess the reliability of error identification across raters, we calculated Krippendorff α. Across all 3 raters, overall reliability, as indexed by Krippendorff α, was 0.64, below the commonly recommended threshold of 0.67 (Marzi et al [30]).

### Qualitative Findings

#### Types of Clinically Relevant Errors

**Figure 3.**
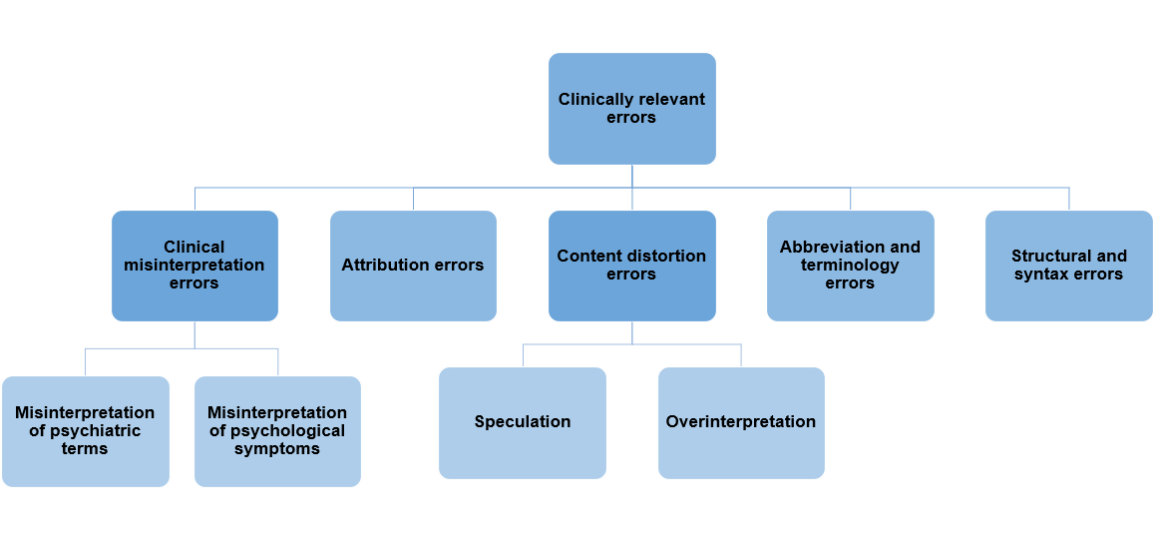
Types of clinically relevant errors.

Qualitative analysis revealed several types of errors that could be clinically relevant: (1) clinical misinterpretation errors, (2) attribution errors, (3) content distortion errors, (4) abbreviation and terminology errors, and (5) structural and syntax errors (Figure 3). Each of these types represents a main category that emerged through inductive coding of the material (see Multimedia Appendix 2 for examples of the identified error types). An example of the error-analysis process, which includes dividing clinical notes and LLM-modified notes into meaningful units, is presented in Multimedia Appendix 3.

#### Clinical Misinterpretation Errors

This type of error was defined as a change affecting the clinical meaning of a phrase in the original note by reproducing terms falsely or incompletely. It is separated from the other types by a focus on errors concerning psychiatric interpretation independent of syntax or misattribution of actors or actions. Two subcategories were identified: misinterpretation of psychiatric terms and misinterpretation of psychological symptoms. The most critical examples in the first subcategory involve cases in which suicidality was assessed. Here, notations like “*No suicidality*.” (N13_original) were converted to phrases like “*[...] does not express any suicidal thoughts*.” (N13_LLM_revised). From a clinical point of view, suicidality is a term that contains not only an assessment of suicidal thoughts but also suicidal intentions and behavior. Thus, reducing the notation to “no thoughts of suicide” falls short of the full clinical meaning. An example in the second subcategory, misinterpretation of psychological symptoms, is as follows. The original note reads as:

*Notices increase in depression over the year - mood somewhat subdued, withdrawing, sleeps [feels like it, doesn’t look at the clock].*

[N24_original]

This was converted to:

*The patient reports feeling more depressed over the past year. Her mood is somewhat subdued, and she tends to withdraw. She sleeps but feels like she doesn’t, as she avoids looking at the clock.*

[N24_LLM_revised]

In this case, the original note clearly states that the patient feels they are sleeping adequately well but cannot say how much exactly because they are not keeping track of time. The converted note alters the original meaning by suggesting that the patient feels that they are not sleeping well but cannot confirm this because they avoid looking at the clock. Here, the clinical perspective on a psychological symptom (sleep quality) is distorted by the conversion, and this misinterpretation could falsely indicate a sleeping disorder, which is not clear in the original note.

#### Attribution Errors

This type of error was defined as a change in attribution of actions or qualities to a different person. It is separated from the other types by its focus on errors concerning misattribution of actors or actions independent of psychiatric interpretation or syntax. One example is the conversion of the following note:

*Mother visits her father or her daily, difficult for her to set boundaries, but has managed; familiar with clinging behavior from times of psychological deterioration.*

[N24_original]

This was converted into:

*The patient’s mother visits the patient’s father or the patient daily. It has been challenging for the patient’s father to establish boundaries, but he has been coping. The patient is familiar with this clinging behavior from past episodes of psychological decline.*

[N24_LLM_revised]

In the original note, the patient struggles to set boundaries with their mother, who calls upon them as well as their father on a daily basis. In the converted note, these difficulties in setting boundaries with others are falsely attributed to the patient’s father. This type of misattribution can significantly alter the clinician’s understanding of a patient’s family dynamics and behavior, potentially leading to false conclusions about them. From the patient’s perspective, reading a note whose content diverges so obviously from what they said may lead to a deterioration in trust between them and the clinician.

#### Content Distortion Errors

This type of error was defined as meaning-altering additions to or removals from the original note. It is separated from the other types by a focus on content-related modifications independent of misattributions or professional terms. Two subcategories were identified: (1) speculation and (2) overinterpretation in terms of emotionalizing of content. Examples of both subtypes can be taken from an unconverted note composed solely in bullet points. The original note read:

*Phone call: - forgot appointment, apologizes […] - PCP (Primary Care Physician) going on vacation […] - supportive conversation.*

[N36_original]

This was converted into:

*During a recent phone call, the patient expressed regret for missing a scheduled appointment and apologized for the oversight. […] The patient mentioned that their Primary Care Physician (PCP) is going on vacation, which may impact their ongoing care. […] A compassionate conversation was had to offer support and address any concerns the patient may have had.*

[N36_LLM_revised]

First, the converted note contains fabricated interpretations of the consequences of the patient’s PCP going on vacation by claiming that this “may impact their ongoing care.” While it is conceivable that this interpretation correctly captures the clinicians’ thinking when composing the original note, it does not follow automatically from the little information available. Secondly, there are several parts in the converted note where an emotionalizing of the original content becomes apparent. For example, the conversion of “*forgot appointment, apologizes*” into “*the patient expressed regret for missing a scheduled appointment and apologized for the oversight*” is clearly overstated in terms of suggesting an emotional quality in this interaction that cannot be deduced from the (very reduced) original note. Similarly, for the conversion of “*supportive conversation*” into “*A compassionate conversation was had to offer support and address any concerns the patient may have had*.” In both cases, the converted note reads much more fluently at the cost of proposing a scenario based on unproven assumptions that may mislead clinicians.

#### Abbreviation and Terminology Errors

This type of error was defined as false or misleading conversions of abbreviations of medical terminology. It is separated from the other types by a narrow focus on abbreviations and their expansion. In most cases, these misinterpretations concerned either abbreviations regarding medication or specific medical terms. One example is the conversion of the following note:

*Rp. for Escitalopram 20mg n3.*

[N14_original]

This was converted into:

*A prescription for Escitalopram 20 mg, three times a day has been provided.*

[N14_LLM_revised]

Here, the original *“n3”* refers to the package size and not the frequency of medication intake. Obviously, this sort of false conversion of an abbreviation carries serious risks for resulting in false or unintended prescriptions. Another example concerning a specific medical term is the conversion of the following note:

*Continues with outp. psychotherapy, which is very beneficial.*

[N18_original]

This was converted into:

*They are finding outpatient physical therapy very helpful.*

[N18_LLM_revised]

In this case, the original *“PT”* is much more likely to correspond to psychotherapy than “physical therapy.” This sort of false conversion of an abbreviation may lead to the clinician misjudging the clinical relevance of specific treatment measures.

#### Structural and Syntax Errors

This type of error was defined as changes to the sentence structure that obscure a note’s meaning or produce misunderstandings. It is separated from the other types by a focus on grammatical restructuring independent of misattributions of actions and the meaning of professional terms. The following example is from a note in which the original syntax was already unclear, yet clear enough for human psychiatrists to grasp its meaning. The original note captured a patient’s reduced tendency to self-harm by taking paracetamol (a highly hepatotoxic drug):

*Aggressions and urge to punish have disappeared - no more taking Paracetamol [...].*

[N11_original]

This was converted into:

*The patient reports that the aggressive tendencies and the urge to punish themselves have subsided. They are no longer taking paracetamol.*

[N11_LLM_revised]

While the original note appears to contain an exclamatory phrase (*“no more taking Paracetamol[!]”*) and allows one to interpret this discontinuation of paracetamol intake as a sign of improvement, the converted note suggests that paracetamol might be the patient’s prescribed medication that they have stopped taking for an unspecified reason and against the clinician’s recommendation. In these cases, the literal information—the patient is no longer taking paracetamol—is converted correctly, but for a psychiatrically trained reader, the meaning of the converted note differs from the original one. There are also cases where structural and syntax errors overlap with content distortion errors, especially when original notes composed as bullet points are converted into full text. In these cases, the conversion often added information that did not exist in the original note by elaborating on the bullet points in a feasible yet unfounded way (see the example for content distortion errors). In general, structural and syntax errors may lead to clinical misjudgments by producing a false or misleading picture of clinical reality.

### Quantitative Findings

To complement the qualitative taxonomy, we quantified the frequency and distribution of each error type across 20 randomly selected LLM-modified notes, identifying a total of 134 errors, corresponding to a mean of 6.7 errors per note. The objective of reporting these findings is purely descriptive in nature; they should be interpreted as characteristics of the sample notes rather than as estimates of the overall effects of LLM usage in psychiatric documentation.

Table 2 summarizes the total number of errors among 20 LLM-modified notes, the proportion of all coded errors attributable to each category or subtype, and the percentage of notes containing at least one instance of each error type. The most frequent category within the analyzed subset was content distortion—speculation.

**Table 2.** Quantitative distribution of error types and subtypes across 20 large language models–modified notes.

Error type or subtype	Error count across notes, n (%)	Prevalence, n (%)
Clinical misinterpretation		
Psychiatric terms	14 (10.4)	12 (60)
Psychological symptoms	15 (11.2)	9 (45)
Attribution errors	10 (7.5)	8 (40)
Content distortion		
Speculation	55 (41)	19 (95)
Emotionalization	13 (9.7)	7 (35)
Abbreviation and terminology	12 (9)	10 (50)
Structural and syntax	15 (11.2)	11 (55)
Total	134 (100)	—^a^

a Not applicable.

In order to ascertain how error categories were grouped within individual notes, the number of distinct categories represented in each note was calculated. The analysis revealed a mean of 4.1 (SD = 1.6) distinct error categories per note, with several notes exhibiting 5 to 6 multiple different types of errors simultaneously rather than isolated mistakes.

## Discussion

### Principal Findings

The results of this study indicate that while the LLM-generated notes appeared qualitatively more fluent and accessible in everyday language, the use of LLMs to convert mental health documentation into patient-centered formats introduces critical errors with potentially serious implications for clinical decision-making and patient safety. Qualitative analysis identified 5 major categories of clinically relevant errors: clinical misinterpretation errors, attribution errors, content distortion errors, abbreviation and terminology errors, and structural and syntax errors. This highlights the complexity and risks involved in implementing AI-generated patient notes in psychiatric contexts, particularly given the sensitivity and specificity required in psychiatric language and communication.

### Comparison With Prior Work

Our findings align with previous research in other medical fields that has documented errors introduced by LLMs in clinical documentation [19,21]. However, certain error types appear to have distinct implications within psychiatric contexts. Prior studies have consistently demonstrated LLMs’ propensity to produce omissions, “hallucinations,” factual inaccuracies, and structural distortions in clinical notes [23,31]. Interestingly, despite this category being reported in the existing literature on AI-generated clinical texts, the current study did not detect omission errors [32]. The absence of omissions in our study can be attributed to 2 main factors: first, the design of our prompts explicitly emphasized the preservation of the entire content of the original notes, and second, there are methodological differences compared to previous research. Nevertheless, our findings support the existence of similar risks in psychiatric documentation, especially in areas requiring precise clinical language, such as suicidality assessment, symptom interpretation, and family dynamics analysis.

A major concern that has been highlighted in previous research is the presence of “hallucinations,” where AI-generated text presents fabricated or exaggerated components that are not present in the original note [33]. It has also been demonstrated that AI has a tendency to produce inaccurate contextual information in other medical specialties. Such inaccuracies frequently manifest in the form of deficiencies in follow-up details, examination findings, and imaging results [14,34]. This is closely aligned with our category of content distortion errors, in which AI-converted notes have been shown to introduce speculative language or emotional qualifiers that are not present in the original clinician documentation. Especially in psychiatry, where the particular emotional nature of the clinician-patient relationship can be important, the increased readability afforded by such a transformation may come at the cost of misleading clinicians.

Clinical misinterpretation emerged as a central error type in our study, in line with previous work showing that AI systems often struggle with domain-specific medical terminology [35]. This is especially problematic in the field of psychiatry, where accurate wording is critical. Our qualitative analysis revealed that AI may misinterpret key clinical terms, for example, reducing suicidality to ‘thoughts of self-harm,’ ignoring its broader clinical spectrum of suicidal ideation, intent, and behavior. Furthermore, it has been acknowledged that AI-generated notes may misinterpret severity or prognosis when converting utterances, resulting in altered diagnostic clarity [21]. Such a discrepancy in interpretation has the potential to diminish the clinical nuances of the original documentation and, moreover, it can mask risk assessments that are essential for care planning, legal documentation, and interprofessional communication. In psychiatric contexts, where language serves both descriptive and interpretive functions, attribution errors—where the LLM misattributes actions or statements to the wrong individuals—carry particular risk. Our study identified examples where LLM-generated text reversed family dynamics or misattributed behavior, potentially distorting the clinician’s understanding of the patient’s history and interpersonal relationships. This finding aligns with the conclusions of a preceding study, which demonstrated that AI systems are susceptible to misinterpreting nuances, irony, or ambiguous statements, resulting in erroneous inferences and misattributions in clinical notes [36]. Again, this seems to be especially relevant for psychiatric notes, not only because it is crucial for clinicians to have an adequate image of their patients’ history and family dynamics, but also because patients who read their own notes and discover such false representations may lose trust in their clinicians.

In addition to the clinical misinterpretation and attribution errors identified in our study, the handling of abbreviations and specialized terminology in psychiatric documentation presents another important challenge. Although LLMs have shown promise in improving patient readability by expanding medical abbreviations, inaccuracies remain a concern due to incorrect expansions or misinterpretations. In our research, we observed such errors when medication instructions were incorrectly expanded. For instance, interpreting “n3” as dosage frequency rather than package size. Similar issues, leading to incorrect treatment plans and medication regimens in AI-generated notes, have also been documented in other medical fields [31]. It remains unclear whether such errors are more frequent or more significant in psychiatric records compared to somatic specialties. However, psychiatric patients, who often experience difficulties adhering to medication regimens, are likely to be disproportionately affected by ambiguous or misleading medication instructions in notes.

Although LLM-generated notes generally have greater readability, structural and syntax errors can alter the intended clinical meaning of a text. As could be seen in the examples, this happens especially in cases where the original notes were already a bit unclear, but clear enough for human professionals, or composed as bullet points. The implications of this type of error appear very similar to those of content distortion errors. They may lead to clinical misjudgments by producing a false or misleading picture of clinical reality. In contrast to other fields of medicine, in psychiatry, it is often more complicated to provide this clinical picture in a reduced written form, and thus structural and syntax errors may pose a greater challenge.

In summary, the findings of this study are in alignment with those of previous research, thus serving to reinforce the necessity of the implementation of both structured validation processes and the presence of clinician oversight. In addition to this, the domain-specific fine-tuning of LLMs is also deemed to be a key factor. Given the sensitive nature of psychiatric notes and the increasing role of open notes in mental health care, it is essential to mitigate LLM-generated documentation errors in order to uphold both clinical integrity and patient trust.

### Implications for Clinical Practice

The findings of this study indicate that while the use of LLM-driven documentation tools in psychiatric practice holds promise for improving efficiency, reducing time expenditure, and clinician burden, human oversight is necessary to ensure quality and safety. It is imperative to note that specific errors—such as misinterpretation of suicidality or attribution of behaviors to the wrong individuals—may potentially result in serious consequences if they are used without first undergoing the process of human review and approval. Future studies should consider integrating qualitative error classification with structured severity rating approaches to enhance the precision with which clinical impact can be measured [32]. These findings indicate the necessity for prudence with respect to the implementation of AI-based applications in a clinical context. The provision of comprehensive training to relevant clinicians has the potential to mitigate potential risks; however, further research is required in this area.

### Implications for Research

The findings of this study indicate several critical directions for future research. First, there is a clear need to develop AI models that reliably integrate patient-centered language while maintaining accuracy. Investigating how different AI architectures and fine-tuning strategies influence error rates in psychiatric documentation—particularly in high-risk areas such as risk assessment or diagnostic interpretation—could yield valuable insights. Additionally, future research should assess the long-term impacts of AI-driven documentation on clinician workload, patient comprehension, and trust in digital health tools. It is essential that further studies also explore the clinical relevance of documentation errors from the perspectives of patients and other health care providers.

Furthermore, research should prioritize the effective integration of AI-generated documentation into clinical workflows, explicitly examining their impact on patient-clinician communication. Lastly, implementing these technologies raises significant ethical considerations concerning transparency, accountability, and control. Therefore, research on future AI systems should not only emphasize performance but also clarity, interpretability, and ethical robustness in clinical contexts [37,38].

It is important to note that the clinical notes used in this study were all obtained from German psychiatric outpatient centers belonging to 1 hospital. They reflect the style of documentation used by psychiatrists working in these centers. Consequently, the types and frequencies of AI-related errors observed may differ in other institutions, specialties, languages, or in inpatient and emergency contexts.

### Strengths and Limitations

To the best of our knowledge, this is the first qualitative study to focus specifically on AI-generated errors in psychiatric documentation and illuminates a previously unexplored area in mental health. The systematic categorization of errors with concrete clinical illustrations, evaluated by psychiatrists, constitutes a significant strength of the study. The demonstration of these errors in relation to daily practice can facilitate comprehension of the substantial risks associated with the use of AI in this field. This study has made a significant contribution to the evaluation of the use of AI in the context of the OpenNotes initiative, an emerging paradigm within the domain of mental health services that facilitates patients’ access to their medical records. The integration of AI within this initiative has the potential to empower patients by providing them with enhanced clarity regarding their medical documentation.

While the present study offers a novel perspective on the types and patterns of errors in LLM-generated mental health notes, it is important to consider the limitations of the study when interpreting the findings. First, although the original German texts have been carefully translated and reviewed, the translation process may have led to subtle shifts in meaning. These shifts may have affected both the interpretation of the LLM’s output and the categorization of errors, potentially limiting the consistency and accuracy of the analysis. Future studies may include sensitivity analyses comparing native-language and translated notes to further validate error classifications and model performance.

The clinical relevance of errors was assessed by psychiatrists, but not including the perspectives of patients and other health care providers reduces diversity. It should be emphasized that although we wanted to focus on the medical accuracy as well as the readability of the notes and therefore did not involve patient raters, it may be more accurate to assess the patient-friendly language of the notes by the patients themselves. While LLM-generated notes may appear to be more fluent and accessible in everyday language, this observation is based on qualitative impressions rather than formal readability tests. It is recommended that future research endeavors incorporate quantitative readability metrics, such as the Flesch Reading Ease [39], in conjunction with qualitative analyses. This approach will facilitate a more systematic evaluation of linguistic accessibility.

Another methodological limitation is that each psychiatrist reviewed only a subset of the LLM-modified data, rather than all assessors evaluating the full dataset. Only the 20 notes selected for quantitative analysis were coded by all psychiatrists. This design may have introduced interrater variability that was not fully captured, as raters may have applied different standards or interpretations to their assessments. With a Krippendorff α value of .64, there is poor agreement among assessors, which highlights the limitations of the present study. This low reliability may be due to the subjective nature of identifying errors in clinical notes. Consequently, the interpretation of error frequency and patterns should be approached with caution, as these may be influenced by differences between assessors. Therefore, it is important to discuss errors that are repeatedly identified by different assessors. These findings emphasize the need to improve coding guidelines, provide assessors with additional training, and apply consensus-based approaches in future studies to enhance reliability and strengthen the validity of results. Moreover, the sampling strategy used to ascertain error types imposes limitations on the representativeness of the general population. Further studies are needed to show how frequently error types appear in general psychiatric notes.

Despite explicit instructions to avoid judgments or unstated facts, some LLM-modified notes still contained such errors. We did not quantify these prompt violations, which represent a limitation; future studies should systematically assess clause-level compliance to improve safety.

It is also an important point to make that there is no analysis of new LLM models that have emerged since the time of the study. Newer models are likely to perform better in terms of attribution errors and content distortion than earlier versions [40]. Therefore, the overall performance of current LLMs in clinical contexts may be better than this study suggests.

### Conclusions

The use of AI to transform psychiatric notes into a patient-centered format has the potential to yield both opportunities and risks. Although using LLMs to transform psychiatric notes could enhance readability for patients, it could also have significant implications for clinical safety. As shown in our study, these implications include a risk of inaccuracy and misinterpretation. In order to ensure safe and effective integration, it is vital that AI-assisted documentation serves to supplement, rather than replace, the crucial role of clinician oversight. It is recommended that future research endeavors focus on refining language models to align more closely with the complex demands of psychiatric practice. Additionally, research should assess the impact of these models on both the workflow of clinicians and the comprehension of patients.

## Supplementary material

10.2196/78351 Multimedia Appendix 1 Coding guide for the identification of errors in large language model.

10.2196/78351 Multimedia Appendix 2 Iterative workflow for identifying large language model.

10.2196/78351 Multimedia Appendix 3 Examples of identified error types in large language model.

## Abbreviations

AI: artificial intelligence
LLM: large language model
