# Supplementary material for: Errors in AI-Transformed Patient-Centered Mental Health Documentation Written by Psychiatrists: Qualitative Pre-Post Study
Source: JMIR Ment Health. 2026 Apr 29;13:e78351. doi: 10.2196/78351 (PMC13128051; doi:10.2196/78351)
Supplement: Multimedia Appendix 1 [file mental-v13-e78351-s001.docx]

**Multimedia Appendix 1. Coding Guide for the Identification of Errors in LLM-generated Notes**

**Purpose of the Coding Guide**

This coding guide was created for evaluators to use in identifying and classifying errors in psychiatric notes modified by LLM tools to enhance transparency, consistency, and reproducibility. The coding process was executed in accordance with a structured, multi-stage, iterative workflow, which was designed to ensure comprehensive identification of LLM-related errors and minimise subjective interpretation.

**General Coding Principles**

Primarily, assessors are required to evaluate the note independently of one another in order to familiarise themselves with the original note, its clinical content and structure. During this preliminary reading, the notes should be divided into meaning units. These are defined as self-contained text segments that convey a single clinical idea, such as a symptom description, diagnostic consideration, treatment-related information, or relevant contextual detail.

In the next stage, coding must be performed separately for each predefined error category. All coding decisions should be based on a comparison of the original text with the text modified by the LLM. For each category, assessors review the entire note from start to finish and directly compare each unit of meaning in the version generated by the LLM with its counterpart in the original clinical note. Once a category is complete, assessors return to the beginning of the note and repeat the same process for the next category. This step-by-step procedure is repeated until all error categories have been evaluated for each note.

Errors or ambiguities that already exist in the original text, such as content-related or structural errors, should be excluded from the coding process. It is important to note that a single note passage may contain multiple error types. It is essential to note that each of these must be coded separately. The original notes written by the clinicians and the modified notes created by the LLM should be first divided into meaning units. Units may be sentences or parts of a sentence. The notes should be then analyzed using a method that looks at each predefined error category. This involves reading the units and the full text again and again. Should the evaluators be uncertain about the category in which an entry should be placed, this issue will be noted and discussed among the evaluators in an effort to reach a consensus. There are a total of 5 main error type groups as identified by Özkara Menekseoglu et al. For concrete examples of each error type see Appendix XX:

**1.Clinical Misinterpretation:** This error should be coded when a change in clinical meaning is observed due to terms in a sentence in the original note being reproduced incorrectly, simplified, or incompletely after LLM modification. This phenomenon can be observed in two distinct groups:

1. Psychiatric Terms
2. Psychological Symptoms

**2. Attribution Errors:** This error type should be coded when, following LLM modification, the relationship dynamics, roles, behaviors, emotions, or actions between patients and third parties (e.g. family members or caregivers) as described in the original text are attributed to individuals other than those defined in the original text.

**3. Content Distortion:** This type of error should be coded when additions not present in the original text, emotional exaggerations, and inappropriate contextual assumptions are encountered after modification of the original text with an LLM.

1. Speculation
2. Emotionalization

**4. Abbreviation and Terminology Errors:** This error should be coded when medical terminology abbreviations in the original text are incorrectly expanded or misinterpreted.

**5. Structural and Syntax Errors:** Errors of this kind are defined as changes occurring within the structure of a sentence subsequent to LLM modification, which serve to render the meaning of the note ambiguous or result in misinterpretation. Errors in the attribution of actions and alterations in the meaning of professional terminology should not be classified as mistakes within this particular category. This category is distinct from other types in that it focuses independently on the restructuring of grammatical structure.
