# Supplementary material for: Errors in AI-Transformed Patient-Centered Mental Health Documentation Written by Psychiatrists: Qualitative Pre-Post Study
Source: JMIR Ment Health. 2026 Apr 29;13:e78351. doi: 10.2196/78351 (PMC13128051; doi:10.2196/78351)
Supplement: Multimedia Appendix 2 [file mental-v13-e78351-s002.docx]

| Multimedia Appendix 2. Examples of Identified Error Types in LLM-Modified Clinical Notes | | | | |
| --- | --- | --- | --- | --- |
| **Error Type** | **Original Excerpt** | **LLM Output** | **Description of Error** | **Clinical Implication** |
| **Clinical Misinterpretation- Psychiatric Terms** | “No suicidality.” | “although the patient does not express any suicidal thoughts.” | narrows original to only thoughts, omitting intent/behavior. | It may incorrectly indicate a difference in suicide risk. |
|  | “no urge to harm oneself” | “no thoughts of self-harm” | While the original phrasing refers specifically to the absence of an urge or impulse, the LLM substitutes this with thoughts, which represent a different clinical construct. | It may misrepresent the patient's risk profile |
|  | “no more taking Paracetamol. Feels a bit strange - but is okay.” | "no longer needs to take Paracetamol. She mentions feeling a bit strange but overall okay.” | At the original note patient reported action or instruction. LLM.modified note implies a clinical recommendation or justification that is not present in the original note | It may risk miscommunicating medical advice. |
| **Clinical Misinterpretation – Psychological Symptoms** | „reflective“ | „recognizes a pattern“; | While “reflective” simply denotes a thoughtful or introspective stance, “recognizes a pattern” implies a specific cognitive insight that was not stated in the original note. | introduces an unwarranted inference about the patient’s cognitive processing. |
|  | "…drive…" | "…motivation…" | In psychiatry, "drive" (psychomotor drive) can refer to energy, initiative, or biologically‑rooted activity levels beyond just motivation, | this substitution risks loss of clinical nuance |
|  | "depressive mood" | “persistant sadness” | While “depressive mood” is a clinical term indicating a symptom within a diagnostic framework, “persistent sadness” introduces duration (“persistent”) and a specific emotional label (“sadness”) that were not stated in the original note. | misrepresent symptom severity or chronicity |
| **Attribution Error** | “he hides … wife” | “her partner … her wife,” | incorrect pronoun substitution and incorrect gender assignment. | Alters clinical understanding of Misassigned pronouns or genders can lead to identity misrepresentation, confusion about who is being described, and potential clinical misunderstandings (e.g., about family structure, relationship dynamics, or safeguarding concerns).dynamics. |
|  | “patient managing boundaries” | “he has managed” | mixing up gender |  |
|  | “Good in contact, able to agree on things, appropriate in behavior, well-groomed” | “You are DOING WELL in our interactions, able to communicate effectively and make decisions” | It adopts second-person (“you”) throughout for patient actions, experiences, and intentions, while original uses third person as specified in professional note style. | Perspective shifts can create ambiguity about who is speaking, blur boundaries between clinician and patient |
| **Content Distortion**  **Speculation** | “possibility of engaging in group activities” (passive) replaces (scheduled action), speculating on intent and agency.; | “search for group activities” | The LLM replaces the original scheduled, concrete action with a passive, hypothetical phrasing. | It may lead to misunderstandings in care coordination and potentially impact follow-up decisions. |
|  | “Sleeps poorly. No suicidality.” | “Sleep is negatively impacted, although the patient does not express any suicidal thoughts”), | Implies a clinical or causal link between poor sleep and suicidality not present in original note. | Fabricating causal relationships may mislead clinicians regarding the patient’s symptomatology |
|  | “struggling to detach from ruminative thoughts. Describes feelings of incompleteness” | “the patient recognizes a pattern of struggling to detach from persistent thoughts, which contributes to a sense of incompleteness” | Original note simply lists without linking them. | It can mislead clinicians about symptom relationships |
| **Content Distortion – Emotionalization** | “Complains about not having anything that truly excites him.” | “expressed longing for activities or experiences that truly excite” | emotionalizes complaint, not simply reporting. | Adding emotional nuance where the original note is neutral may bias clinicians’ perception of the patient’s affect or motivation |
| **Abbreviation / Terminology** | “Med. for ongoing treatment” | “The patient will continue their ongoing medical treatment.” | “Med. for ongoing treatment” almost certainly refers to “medication,” not “medical treatment,” losing specificity and risking misunderstanding. | It may mislead the patient about the treatment plan. |
|  | “Rp” | (this content has been excluded) |  |  |
| **5. Structural / Syntax** | “Benchmark for adolescent dot > compared to siblings ” | “Additionally, a benchmark for the patient's adolescent daughter was established, comparing her to her siblings.” | Original note presents much information in bullet points and short fragments, which in clinical settings concisely record discrete issues. LLM-modified note restructures content into connected, fluid sentences, sometimes forming causal or emotional relationships where none existed. | This grammatical restructuring can obscure the original meaning by over-connecting or elaborating symptoms, actions, or patient history. |
