# Supplementary figures and images for: Errors in AI-Transformed Patient-Centered Mental Health Documentation Written by Psychiatrists: Qualitative Pre-Post Study
Source: JMIR Ment Health. 2026 Apr 29;13:e78351. doi: 10.2196/78351 (PMC13128051; doi:10.2196/78351)

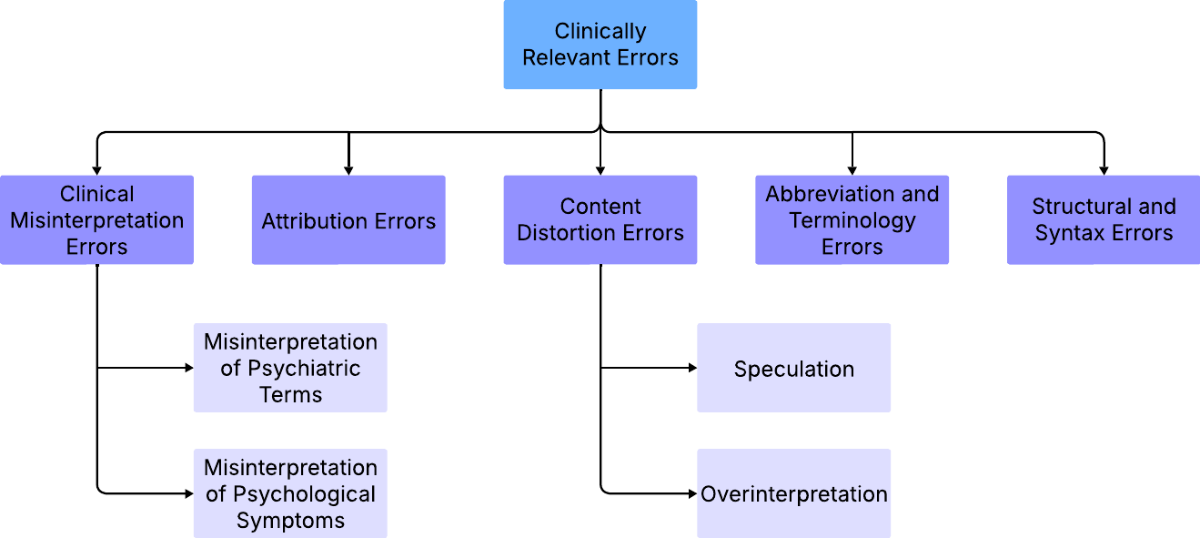

Supplement: Multimedia Appendix 3 [file mental-v13-e78351-s003.png]
